# Supplementary material for: Functional convalescent plasma antibodies and pre-infusion titers shape the early severe COVID-19 immune response
Source: Nat Commun. 2021 Nov 25;12:6853. doi: 10.1038/s41467-021-27201-y (PMC8617042; doi:10.1038/s41467-021-27201-y)
Supplement: Supplementary file 1 — Supplementary Information [file 41467_2021_27201_MOESM1_ESM.pdf]

Supplementary Information

Functional Convalescent Plasma Antibodies and Pre-Infusion Titers Shape Early Severe COVID-19 Immune Response

Jonathan D. Herman, Chuangqi Wang, Carolin Loos, Douglas Lauffenburger, Liise-anne Pirofski,
Galit Alter

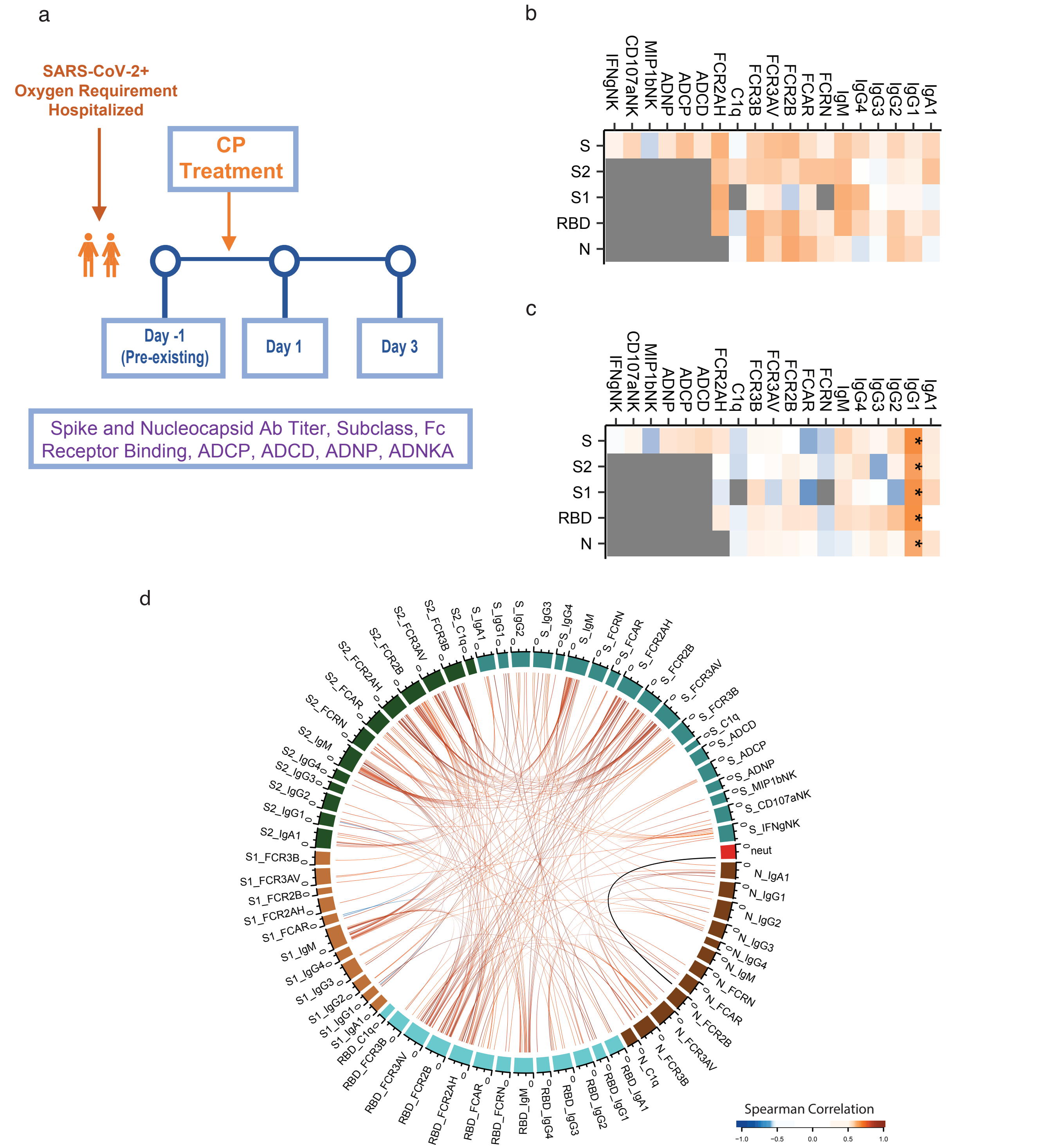

Supplementary Figure 1. Design of Study and Correlation of CP Features.
(A) A diagram of the open label single arm trial of COVID-19 CP administered via the Expanded Access Protocol at Montefiore Medical Center in hospitalized patients who were SARS-CoV-2 positive and had an oxygen requirement. Serum samples from patients were taken prior to CP administration (Day -1), one day after CP administration (Day 1), and three days after CP administration (Day 3). The Day -1, Day 1, Day 3 serum samples and CP units administered to the patients were analyzed for Spike and Nucleocapsid titer, subclass, Fc-receptor binding level, and Ab functions. **(B, C)** Correlation of Neutralizing antibody titer **(B)** and Spike IgG1 titer **(C)** with Spike, S1, S2, RBD, and Nucleocapsid-specific features. An asterisk represents a statistically significant correlation after multiple-test correction. Grey boxes represent assays that were not performed. **(D)** A cord diagram representing the Spearman correlation >0.75 among all SARS-CoV-2 antibody features in CP units. The strength of the correlation is represented by the color of the cord connecting the two nodes, with one exception, correlations with neutralizing antibody titer that are colored in black. The width of antibody each feature represents the accumulated values of Spearman correlation coefficients of that feature with all other features included in the diagram. Source data are provided as a Source Data file.

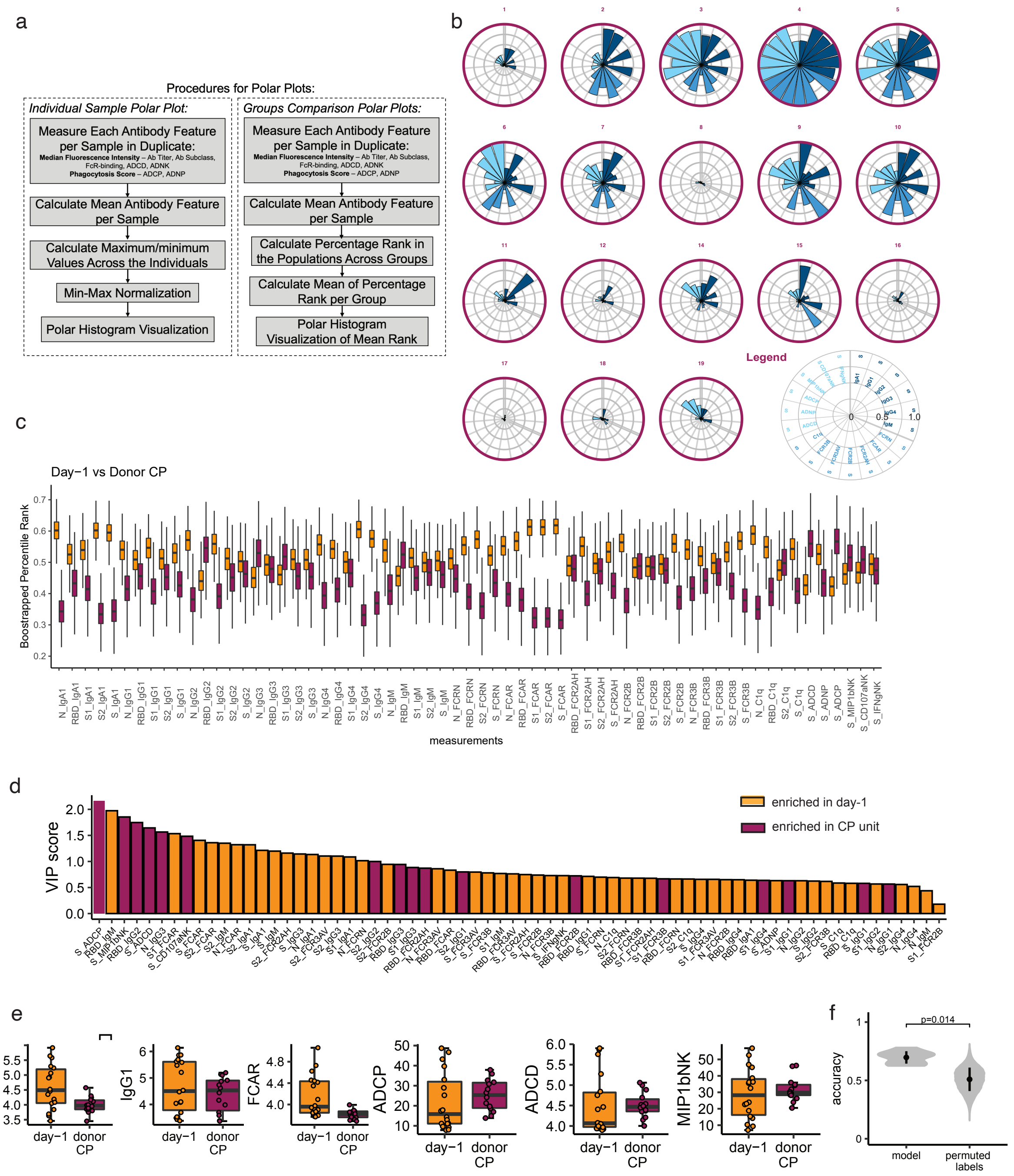

Supplementary Figure 2.
Supplementary figures supporting **Figure 2**, comparing the SARS-CoV-2 Specific antibody responses in CP units vs. Recipients prior to CP (day -1).
(A) Flow diagram of the data processing steps to make individual sample polar plots and group comparison polar plots. **(B)** Day -1 CP recipients were profiled for SARS-CoV-2 S-specific antibody responses. Each polar plot depicts an individual CP recipient's anti-Spike antibody profile, scaled to the minimum and maximum of the 14 units of CP profiled. Each wedge represents a SARS-CoV-2 antibody feature, and the size of the wedge indicates the magnitude of the value. The colors represent the feature group: dark blue - antibody isotypes and subclasses; blue – Fc-receptor binding levels; light blue – antibody-dependent functions; light grey - neutralizing antibody titer. **(C)** To demonstrate the robustness of the polar plots in Figure 2a, we bootstrapped the patients ab-features and calculated the mean percentile in Day-1 and Donor CP for 1000 times. The box plot graph demonstrates the variance of the mean of percentile rank of the SARS-CoV2-Specific Ab features in CP recipient day -1 (n=18) plasma and CP units (n=14) for the antigens N, RBD, S1, S2, and S displayed in the Figure 2B radial plots with a stratified bootstrapped sampling approach. Each box represents the median (central line) and IQR (25% and 75% percentiles) and the two whiskers represent 1.5 * IQR. **(D)** Variable importance in projection (VIP) scores for all 81 antibody features included in the PLS-DA model in **Figure 2 B, C**. The color of the bar indicates in which group the feature is enriched, i.e., has a higher median value. **(E)** Boxplots showing examples of S-specific antibody features for patient samples at day -1 (n=18) and CP units (n=14). IgA1, IgG1, FCAR, and ADCD are reported as log10 MFI values, ADCP as phagocytosis score, and ADNK measured as MIP-1β NK, the percentage of NK cells positive for MIP-1β. Box plots represent the interquartile range, whiskers represent 1.5* the IQR, and the median is indicated by a central line. **(F)** Accuracies from 10 repetitions of five-fold cross-validation for the actual model and models based on permuted labels. The permuted labels models are repeated 500 times for each cross-validation repetition. The two-sided p-values are determined based on the probability that the accuracy of the permuted label model is higher than for the actual model and is reported as median of 10 cross-validation repetitions. The model achieved a median accuracy of 72%. The violin plot represents the distribution of p-values and the whiskers represent 1 standard deviation**.** Color of the box plots **(C), (D), (E)** represents the clinical group: yellow and maroon and represents CP recipient day -1 and CP units respectively. Source data are provided as a Source Data file.

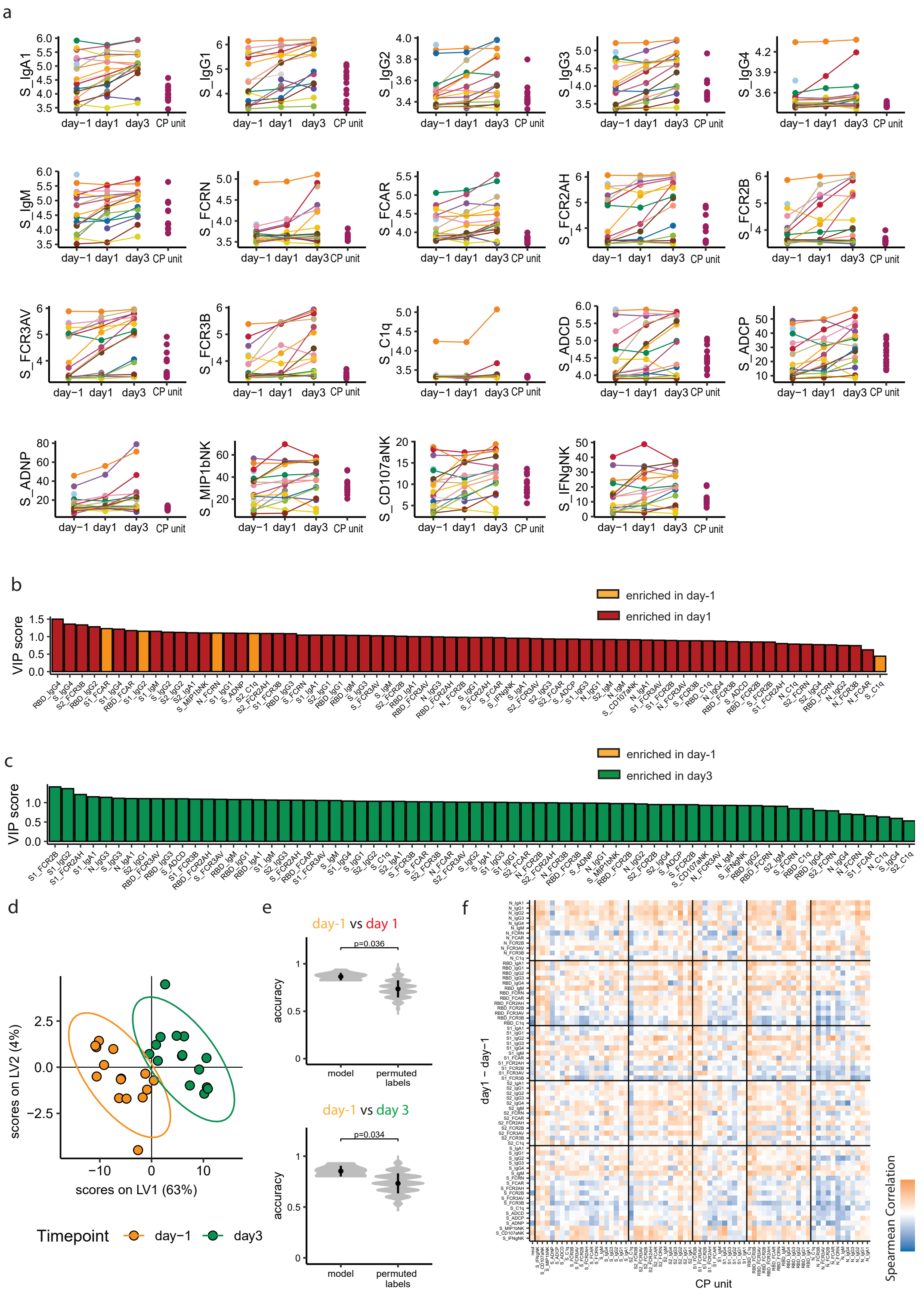

Supplementary Figure 3.

Additional figures supporting **Figure 3**, demonstrating the difference in SARS-CoV-2 specific antibody responses between CP recipient day -1 and CP, and CP recipient day -3 and CP.

(A) SARS-CoV-2 S-specific antibody features for each of the 19 patients and 14 CP units. **(B,C)** Variable importance in projection (VIP) score plot for the 81 antibody features used to construct the mPLS-DA. The color of the bar indicates in which group the feature is enriched, i.e., has a higher median value. (B) day -1 vs. day 1 mPLS-DA model. (C) day -1 vs. day 3 mPLS-DA model. **(D)** Multi-level partial least squares discriminant analysis (mPLS-DA) scores plot for the first two latent variables for the day -1 vs. day 3 mPLS-DA model. Each dot is one sample, and the ellipses indicate 95% confidence regions assuming a multivariate t distribution. Colors indicate the time point the samples were taken for the n=15 patients. The day -1 vs. day 3 model achieved an average cross-validation accuracy of 86%. **(E)** Accuracies from 10 repetitions of five-fold cross-validation for the actual model and models based on permuted labels. The permuted labels models are repeated 500 times for each cross-validation repetition. The two-sided p-values are determined based on the probability that the accuracy of the permuted label model is higher than for the actual model and is reported as the median of 10 cross-validation repetitions. The violin plot represents the distribution of p-values and the whiskers represent 1 standard deviation. **(F)** Heatmap showing the Spearman correlation coefficients between increases in antibody levels between day -1 and day 1 and corresponding CP unit antibody levels. Source data are provided as a Source Data file.

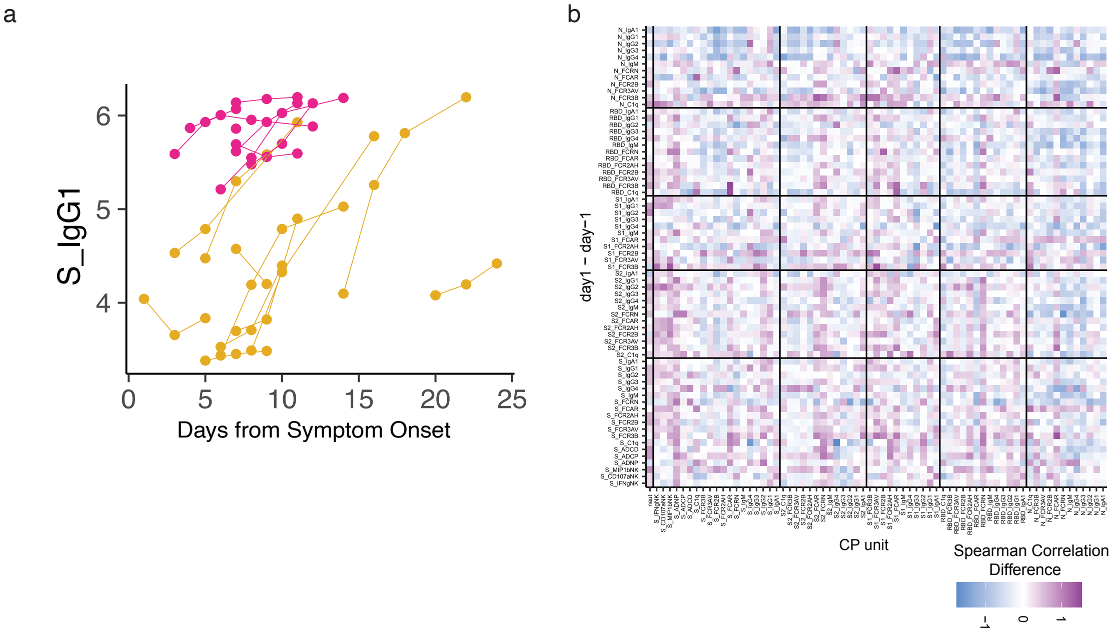

Supplementary Figure 4.

Additional figures supporting **Figure 4**, demonstrating the differences in the effect of CP on the evolution of the anti-SARS-CoV-2 humoral immune response dependent on pre-existing S IgG1 titers.

(A) SARS-CoV-2 S-specific IgG1 titers for each of the 19 patients were arranged by days from COVID-19 symptom onset. Patients with high pre-existing S-IgG1 titers are colored in pink and patients with low pre-existing S-IgG1 titers are colored in mustard yellow. **(B)** The heatmap depicts the difference of the Spearman correlations (CP unit vs. CP recipient day 1 – day-1 plasma) between individuals with high or low S-IgG1. The color represents the strength of the difference in correlation with purple and blue colors representing high and low differences respectively between the two groups. Source data are provided as a Source Data file.

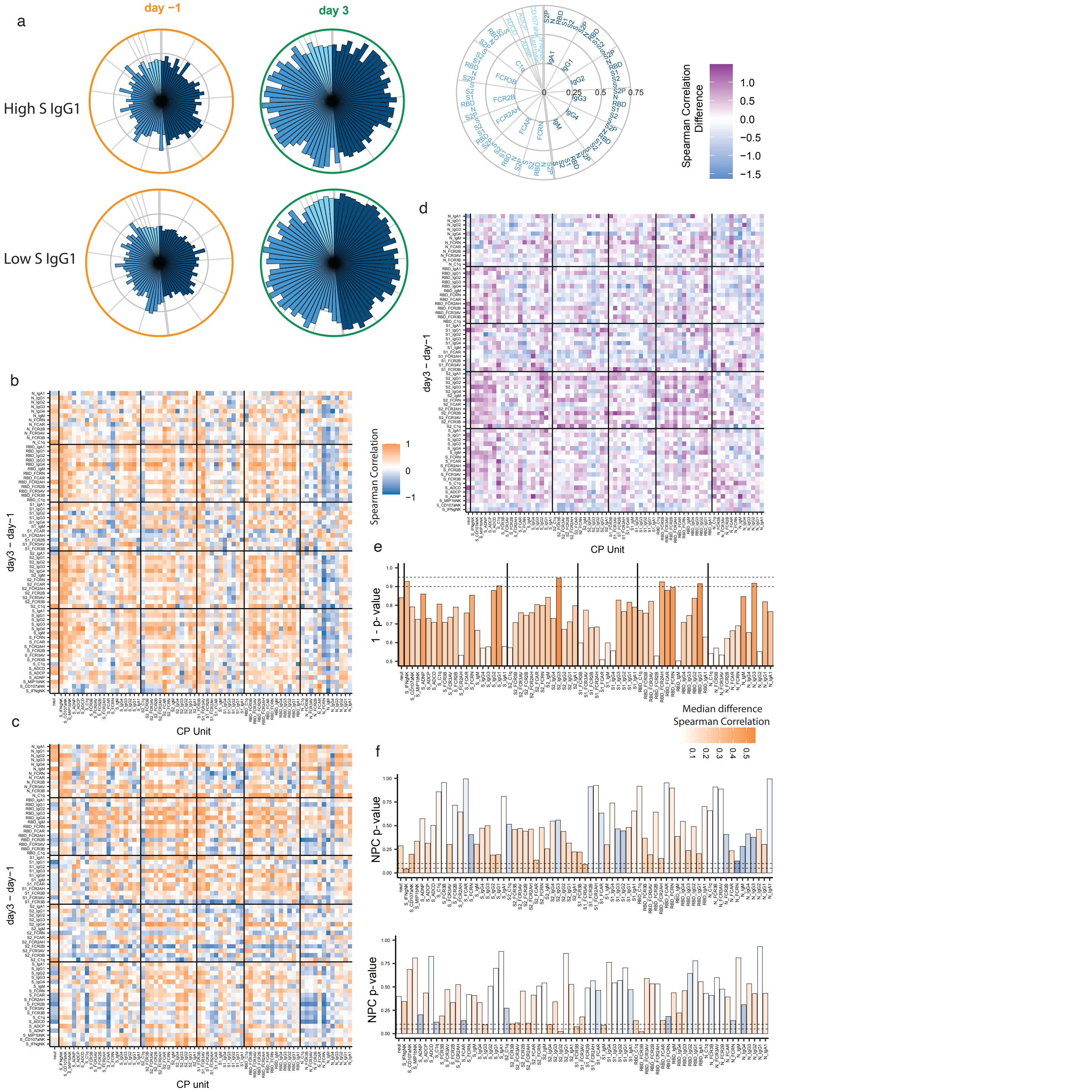

Supplementary Figure 5.

The effect of CP antibody features on the trajectory (day 3 – day -1) of the SARS-CoV-2 humoral response was profiled separately for CP recipient with high and low pre-existing (day -1) S-IgG1 titers.

(A) The polar plots depict the mean percentiles of SARS-CoV-2-specific antibody features within the day -1 (n=18) and day 3 (n=16) groups for the antigens N, RBD, S1, S2, and S in the High and Low Spike IgG1 groups. Each wedge represents a SARS-CoV-2 antibody feature, and the size of the wedge indicates the magnitude of the value. The colors represent the feature group: dark blue - antibody isotypes and subclasses; blue – Fc-receptor binding levels; light blue – antibody functions. **(B, C)** The heatmap shows the Spearman correlation coefficients between increases in antibody levels between day 3 and day -1 (y-axis) and corresponding CP unit antibody features (x-axis) in patients with high **(B)** and low **(C)** levels of pre-existing (day -1) S-IgG1 antibody. The color represents the strength of the median Spearman correlation. (**D**) The heatmap depicts the difference of the Spearman correlation (CP unit vs. CP recipient day 3 – day-1 plasma) between the high group and low S-IgG1 groups. Stronger differences are indicated by purple and weaker differences are indicated by blue. **(E)** The bar plot shows the statistical significance by permutation testing of the median difference between CP features and patient trajectories in High and Low pre-existing IgG1 Spike patients. The Color represents the absolute value of the median difference and the height of the bar represents the two-sided p-value determined by the permutation test. **(F)** Bar plots illustrate the non-parametric combination (NPC) test on each CP unit feature in High pre-existing (D-1) S-IgG1 (top) and Low pre-existing (day -1) S IgG1 (bottom) individuals. The color represents the strength of the median Spearman correlation of each CP feature in the respective High and Low pre-existing (day -1) S-IgG1 titer groups. Source data are provided as a Source Data file.

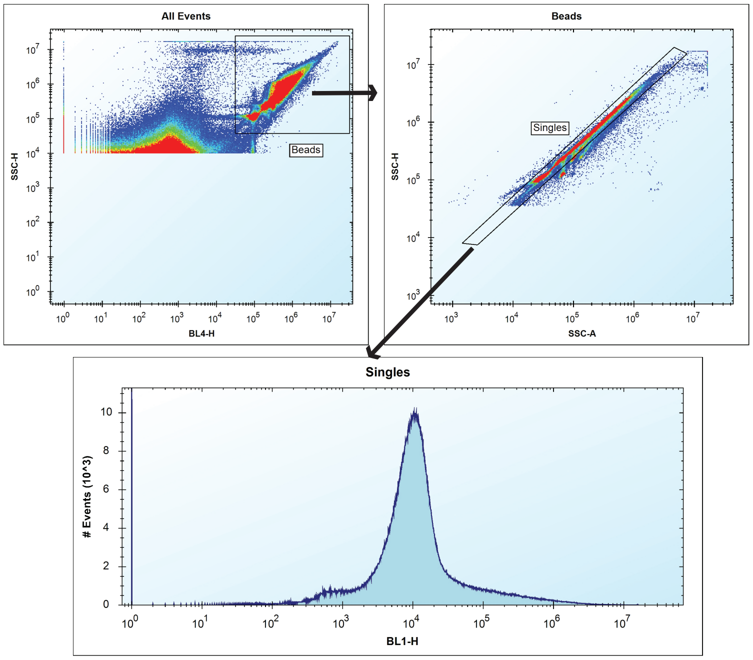

Supplementary Figure 6.

This diagram shows flow cytometry gating strategy for Antibody-dependent Complement Deposition (ADCD). First, red polystyrene beads were selected based on Side Scatter Hight (SSC-H) and fluorescence at 675nm (BL4), next single beads were selected based on SSC-H and Side Scatter Area (SSC-A), and last the median of fluorescence at 530nm (BL1) of single beads was measured.

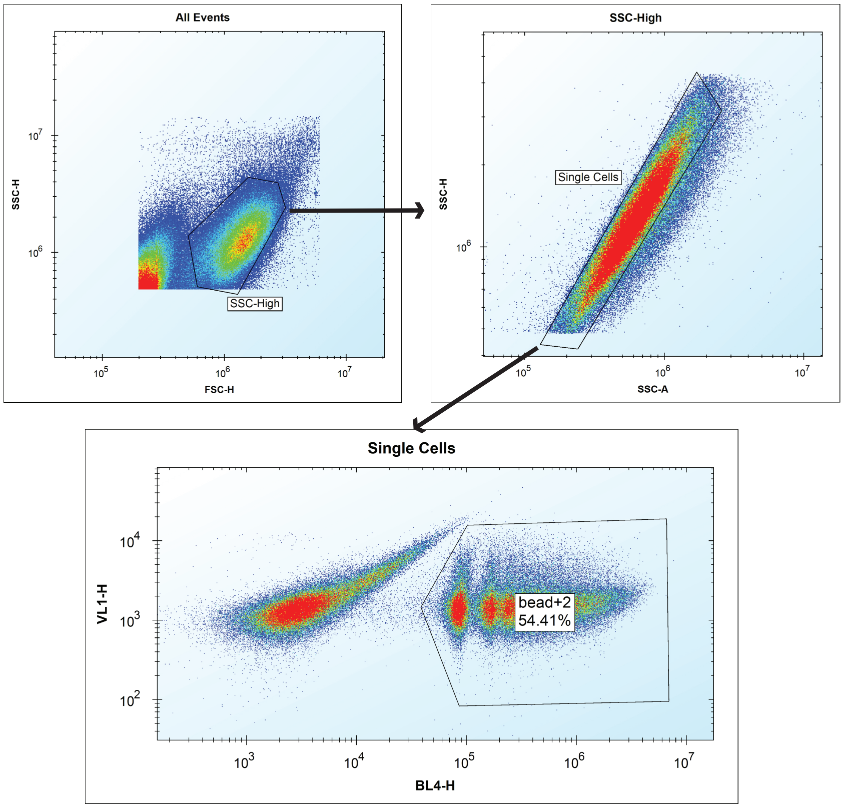

Supplementary Figure 7.

This diagram shows flow cytometry gating strategy for Antibody-dependent Cell Phagocytosis (ADCP). First cells were selected based on SSC-H and FSC-H, next single cells were selected by SSC-H and SSC-A, next cells that had phagocytosed the fluorescent beads were selected based on 675nm fluorescence (BL-4), and lastly a phagoscore was calculated based upon these bead+ single cells.

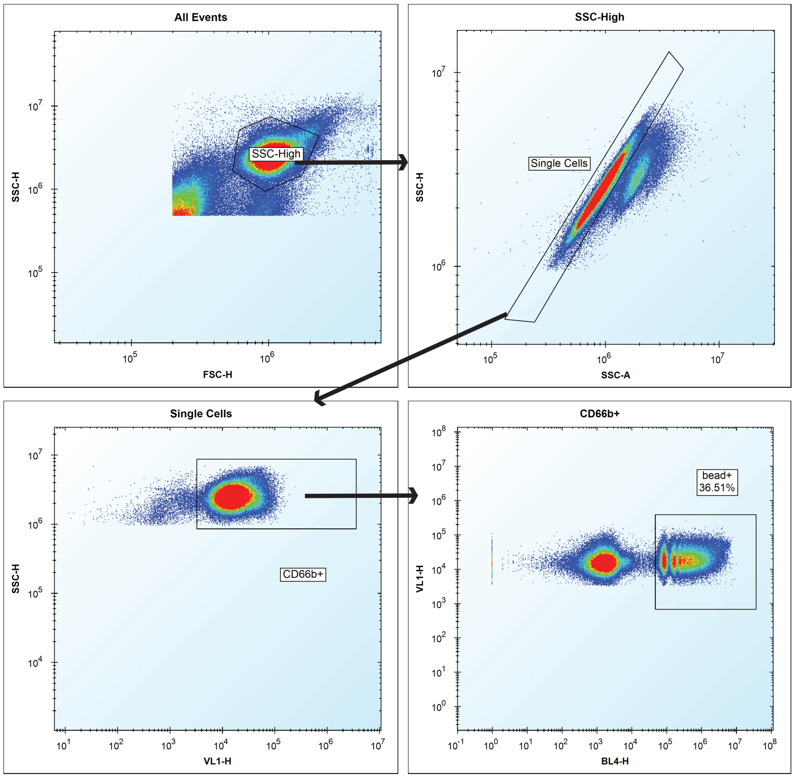

Supplementary Figure 8.
This diagram shows the flow cytometry gating strategy for Antibody-dependent Neutrophil Phagocytosis (ADNP). First cells were selected based on SSC-H and FSC-H, next single cells were selected by SSC-H and SSC-A, next neutrophils were selected based on CD66b-Pacific Blue staining (445nm, VL1), neutrophils that had phagocytosed fluorescent beads were selected based on 675nm fluorescence (BL4), and lastly a phagoscore was calculated based upon these bead+ neutrophils.

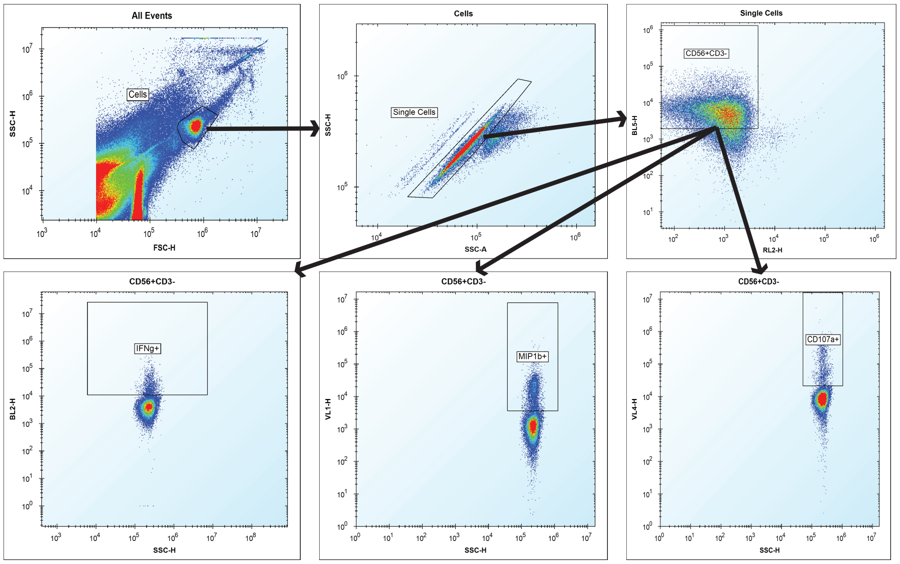

Supplementary Figure 9.
This diagram shows flow cytometry gating strategy for Antibody-dependent NK Cell Activation (ADNK). First cells were selected based on SSC-H and FSC-H, next single cells were selected by SSC-H and SSC-A, and NK were selected based on CD56-PE-Cy7 staining (780nm, RL-2) and absence of CD3-APC-Cy7 staining (445nm, VL1). Interferon-γ producing NK cells were selected based on IFNγ-PE staining (572nm, BL2). MIP1b producing NK cells were selected based on MIP1b-BV421 staining (445nm, VL1). NK cells that had degranulated were selected based on surface staining with CD107a-BV605 (615nm, VL4).

Supplemental Table 1. Clinical characteristics of participants who received CP through the Expanded Access Protocol at Montefiore Medical Center in the Bronx, NY as described in Yoon, et al.

**Baseline patient characteristics**	**CP recipients (n=19)**
Age, median (IQR) - yr	61 (53 – 77)
Sex, male – no. (%)	10 (53)
BMI[1], median (IQR)	27 (23 – 30)
Ethnicity[2] – no. (%)	
Hispanic	9 (47)
Non-Hispanic	9 (47)
Unknown	1 (5)
Race[2] – no. (%)	
White	2 (11)
African American	5 (26)
Asian	2 (11)
Other/Not reported	10 (53)
Comorbidities – no. (%)	
Hypertension	15 (79)
Diabetes	8 (42)
Chronic lung disease	3 (16)
Chronic kidney disease	7 (37)
Coronary artery disease	1 (5)
Heart failure	3 (16)
Hyperlipidemia	12 (63)
**COVID-19 treatments administered during study – no. (%)**	
Corticosteroids	17 (89)
Methylprednisolone	13 (68)
Hydrocortisone	4 (21)
Dexamethasone	3 (16)
Prednisone	9 (47)
Hydroxychloroquine	9 (47)
Remdesivir	1 (5)
Sarilumab	1 (5)
Leronlimab	1 (5)
**Corticosteroids treatment characteristics**	
Corticosteroids given after CP – no. (%)	2 (10)
Total corticosteroids dose over admission, median methylprednisolone-equivalent mg (IQR)	288 (192 – 384)
Days of corticosteroids therapy, median (IQR)	5 (4 – 7)
Average daily corticosteroids dose, median methylprednisolone-equivalent mg (IQR)	54 (48 – 63)
**Clinical characteristics of participants**	
Days between symptom onset and transfusion, median (IQR)	7 (6 – 8)
Hospital day CP administered, median (IQR)	1 (1 – 2)
11-point WHO score at day 0[3] – no. (%)	
5 (Hospitalized, oxygen by mask or nasal prong)	10 (53)
6 (Hospitalized, oxygen by NIV or HFNC)	4 (21)
8, 9 (Hospitalized, MV)	5 (26)
11-point WHO score at day 28 – no. (%)	
0-3 (Not hospitalized)	13 (68)
6 (Hospitalized, oxygen by NIV or HFNC)	1 (5)
10 (Death)	5 (26)
Hospital day of discharge, median (IQR)	9 (7 – 14), n=14
Hospital day of death, median (IQR)	14 (7 – 14), n=5

[1]BMI is calculated as weight in kilograms divided by height in meters squared.

[2]Information on race and ethnic group was obtained from entries in the medical record, as reported by the patients.

[3]Day 0 is the day convalescent plasma was transfused.

Abbreviations: BMI, body-mass index; COVID-19, Coronavirus disease 2019; CP, convalescent plasma; HFNC, high flow nasal cannula; IQR, interquartile range; MV, mechanical ventilation; NIV, non-invasive ventilation; WHO, World Health Organization.
