# Supplementary material for: Functional convalescent plasma antibodies and pre-infusion titers shape the early severe COVID-19 immune response
Source: Nat Commun. 2021 Nov 25;12:6853. doi: 10.1038/s41467-021-27201-y (PMC8617042; doi:10.1038/s41467-021-27201-y)
Supplement: Supplementary file 3 — Reporting Summary [file 41467_2021_27201_MOESM3_ESM.pdf]

Corresponding author(s): Alter, Galit

Last updated by author(s): Oct 23, 2021

Reporting Summary

Nature Portfolio wishes to improve the reproducibility of the work that we publish. This form provides structure for consistency and transparency in reporting. For further information on Nature Portfolio policies, see our Editorial Policies and the Editorial Policy Checklist.

Statistics

For all statistical analyses, confirm that the following items are present in the figure legend, table legend, main text, or Methods section.

n/a	Confirmed	
☐	☒	The exact sample size (*n*) for each experimental group/condition, given as a discrete number and unit of measurement
☐	☒	A statement on whether measurements were taken from distinct samples or whether the same sample was measured repeatedly
☐	☒	The statistical test(s) used AND whether they are one- or two-sided<br>*Only common tests should be described solely by name; describe more complex techniques in the Methods section.*
☐	☒	A description of all covariates tested
☐	☒	A description of any assumptions or corrections, such as tests of normality and adjustment for multiple comparisons
☐	☒	A full description of the statistical parameters including central tendency (e.g. means) or other basic estimates (e.g. regression coefficient) AND variation (e.g. standard deviation) or associated estimates of uncertainty (e.g. confidence intervals)
☐	☒	For null hypothesis testing, the test statistic (e.g. *F*, *t*, *r*) with confidence intervals, effect sizes, degrees of freedom and *P* value noted<br>*Give P values as exact values whenever suitable.*
☒	☐	For Bayesian analysis, information on the choice of priors and Markov chain Monte Carlo settings
☐	☒	For hierarchical and complex designs, identification of the appropriate level for tests and full reporting of outcomes
☐	☒	Estimates of effect sizes (e.g. Cohen's *d*, Pearson's *r*), indicating how they were calculated

Our web collection on statistics for biologists contains articles on many of the points above.

Software and code

Policy information about availability of computer code

Data collection	Forecyt Standard Edition 8.1 was used to collect luminex, ADCD, ADCP, ADNP, and ADNK assay data.
Data analysis	R version 4.0.2 was used to generate figures and perform data analysis. Custom code that was generated in available at https://zenodo.org/record/5527197#.YU42RrhKiUl. Specifically, PLS-DA models were generated with the R package 'ropls' (1.22.0) interfaced by R package 'systemsseRology.'(https://github.com/LoosC/systemsseRology). Spearman correlation was performed with the R function 'cor.test.' Cord diagrams were created with 'chordDiagram' in R package 'circlize'(0.4.12). Correlation networks were created using the function 'ggraph' in the R package 'ggraph' (2.0.4).

For manuscripts utilizing custom algorithms or software that are central to the research but not yet described in published literature, software must be made available to editors and reviewers. We strongly encourage code deposition in a community repository (e.g. GitHub). See the Nature Portfolio guidelines for submitting code & software for further information.

Data

Policy information about availability of data

All manuscripts must include a data availability statement. This statement should provide the following information, where applicable:

- Accession codes, unique identifiers, or web links for publicly available datasets
- A description of any restrictions on data availability
- For clinical datasets or third party data, please ensure that the statement adheres to our policy

The dataset generated during and/or analyzed during the current study have been made available in the Supplementary material as a supplemental data file and as a Source data file. No data was stored externally.

Field-specific reporting

Please select the one below that is the best fit for your research. If you are not sure, read the appropriate sections before making your selection.

☒ Life sciences ☐ Behavioural & social sciences ☐ Ecological, evolutionary & environmental sciences

For a reference copy of the document with all sections, see nature.com/documents/nr-reporting-summary-flat.pdf

Life sciences study design

All studies must disclose on these points even when the disclosure is negative.

Sample size

No sample size calculation was performed. The cohort analyzed in this study consisted of 19 severely ill COVID-19 patients, who received CP within 72 hours of hospital admission from April 13th to May 4th 2020 in the Bronx, NY, and the CP units they received. This single arm unblinded study was conducted under the Mayo Clinic's Convalescent Plasma Expanded Access program. The 19 patients were selected based on availability of plasma for the three time points (Day -1, Day 1, and Day 3) in the convalescent plasma cohort published in Yoon et al.

Data exclusions

Antibody features were excluded from the analysis if the maximal value across samples and time points (day -1, day 1, day 3 and donor CP) was less than four standard deviations above the mean value obtained for PBS controls. Three features (N_FCR2AH, S1_FCRN, S1_C1q) were excluded from the analysis in all of the primary text and supplementary figures. This data exclusion was made after data collection. All excluded data is available in the accompanying source data document.

Replication

All antibody feature measurements were carried out in technical duplicate. Correlation among the duplicate of the biophysical and functional assays were performed to ensure reproducibility.

Randomization

As a single arm unblinded study of COVID-19 Convalescent plasma, there was no randomization or control arm. Thus, we cannot differentiate the effect of CP on clinical outcomes or to control for the natural evolution of the humoral immune response, acknowledge this in the manuscript, and focus on other parts of the humoral immune response.

Blinding

As a single arm unblinded study of COVID-19 Convalescent plasma, there was no blinding of the patient populations. During data collection of antibody profiles, the investigators were blinded to the sample identity and any relationships between samples (e.g. CP unit vs. CP recipient day -1, vs. CP recipient day 1, vs. CP recipient day 3). During, data analysis, investigators were unblinded to sample identification to allow for statistical analysis and figure making. Data analysis would not have been possible without sample unblinding.

Reporting for specific materials, systems and methods

We require information from authors about some types of materials, experimental systems and methods used in many studies. Here, indicate whether each material, system or method listed is relevant to your study. If you are not sure if a list item applies to your research, read the appropriate section before selecting a response.

Materials & experimental systems

n/a	Involved in the study	
☐	☒	Antibodies
☐	☒	Eukaryotic cell lines
☒	☐	Palaeontology and archaeology
☒	☐	Animals and other organisms
☐	☒	Human research participants
☒	☐	Clinical data
☒	☐	Dual use research of concern

Methods

n/a	Involved in the study	
☒	☐	ChIP-seq
☐	☒	Flow cytometry
☒	☐	MRI-based neuroimaging

Antibodies

Antibodies used

1 .Mouse Anti-Human IgG1-PE (Southern-Biotech, #9054-09, clone:HP6001) used at a 1:200 dilution.
2 .Mouse Anti-Human IgG2-PE (Southern-Biotech, #9070-09, clone: HP6002) used at a 1:100 dilution.
3. Mouse Anti-Human IgG3-PE (Southern-Biotech, #9210-09, clone:HP6050) used at a 1:100 dilution.
4. Mouse Anti-Human IgG4-PE (Southern-Biotech, # 9200-09, clone: HP6025) used at a 1:100 dilution.
5. Mouse Anti-Human IgM-PE (Southern-Biotech, #9020-09, clone:SA-DA4) used at a 1:100 dilution.
6. Mouse Anti-Human IgA1-PE (Southern-Biotech, #9130-09, clone: B3506B4) used at a 1:100 dilution.
7. Anti-guinea pig complement C3 goat IgG fraction (MP Biomedical, #855385. polyclonal) used at a 1:100 dilution.
8. anti-human CD66b Pacific Blue (Biolegend, #305112 ,clone G10F5) used at a 1:80 dilution.
9. Anti-CD107a BV605 antibody (Biolegand, #328634, clone H4A3) used at a 1:200 dilution.
10. Anti- CD3 APC-Cy7 (BioLegend, #300426, clone UCHT1) used at a 1:160 dilution.
11. Anti-CD56 PE-Cy7 (BD Biosciences, #335791, clone NCAM16.2) used at a 1:80 dilution.
12. Anti-MIP-1ß-BV421 (BD Biosciences, #562900, clone D21-1351) used at a 1:160 dilution.
13. Anti-IFNγ-PE (BioLegend, #506507, clone B27) used at a 1:40 dilution.

March 2021

Validation	Anti-guinea pig complement C3 goat IgG fraction was validated as reactive with guinea pig complement C3 by the manufacturer and validated in a previous manuscript with flow cytometry and ImageStream analysis of Guinea Pig C3-conjugated beads.<br><br>Anti-human CD66b Pacific Blue (Biolegend, #305112 ,clone G10F5) was validated by the manufacturer for staining of Human peripheral blood granulocytes by flow cytometry and in the Alter lab for the staining of purified primary Human neutrophils by flow cytometry.<br><br>Anti-CD107a BV605 antibody (Biolegand, #328634, clone H4A3) was validated by the manufacturer by flow cytometry to stain thrombin-activated human peripheral blood platelets. It was also validated in the Alter lab for staining of PMA/Ionomycin-activated primary Human NK cells activated by flow cytometry.<br><br>Anti- CD3 APC-Cy7 (BioLegend, #300426, clone UCHT1) was validated by the manufacturer for staining of human peripheral blood lymphocytes by flow cytometry. It was validated in the Alter lab for the absence of staining of purified primary Human NK cells by flow cytometry.<br><br>Anti-CD56 PE-Cy7 (BD Biosciences, #335791, clone NCAM16.2) was validated by the manufacturer for staining of CD3- peripheral blood cells. It was validated in the Alter lab for staining of purified primary Human NK cells by flow cytometry.<br><br>Anti-MIP-1ß-BV421 (BD Biosciences, #562900, clone D21-1351) was validated by the manufacturer to stain human peripheral blood mononuclear cells activated with IFN-gamma. It was validated in the Alter lab for staining of PMA/Ionomycin-activated purified primary Human NK cells by flow cytometry.<br><br>Anti-IFNγ-PE (BioLegend, #506507, clone B27) was validated by the manufacturer for staining of PMA/Ionomycin-stimulated BALB/c CD3+ T-cells. It was validated in the Alter lab for staining of PMA/Ionomycin-activated purified primary Human NK cells by flow cytometry.

Eukaryotic cell lines

Policy information about cell lines

Cell line source(s)	THP1 were obtained from ATCC cat# TIB-202, Vero-E6 were obtained from ATCC cat# CRL-1586.
Authentication	Both cell lines were obtained from ATCC. THP1 were validated for surface staining of monocyte-specific markers including Fc receptors and for phagocytic behavior, but not by any genetic means. Vero E6 Cells were obtained from ATCC and were not otherwise authenticated.
Mycoplasma contamination	Mycoplasma negative.
Commonly misidentified lines<br>(See ICLAC register)	None

Human research participants

Policy information about studies involving human research participants

Population characteristics	The cohort consisted of 19 severely ill COVID-19 patients, who received CP within 72 hours of hospital admission from April 13th to May 4th 2020 in the Bronx, NY, and the CP units they received were profiled in this study. Sex breakdown of the study was 53% male and 47% female. The median age of the cohort was 61 years old. All patients required non-invasive oxygen supplementation. At the time of study enrollment, they had a median score of 5 on the 11-point World Health Organization Ordinal Scale for clinical improvement, indicating they were hospitalized and required non-invasive ventilation or high-flow nasal cannula. However, 48% subsequently required non-invasive positive pressure ventilation or mechanical ventilation during the course of this study.
Recruitment	Described in more detail in Yoon et al., adult hospitalized patients with PCR-confirmed COVID-19 were enrolled in the Mayo Clinic Expanded Access Treatment Protocol to receive CCP between April 13 and May 4, 2020. Hospitalized patients were referred to the study team by hospitalists and/or infectious diseases consultants and were deemed eligible to receive CCP if they had been hospitalized for 3 days or were symptomatic for 3 to 7 days prior to transfusion and had severe and/or life-threatening COVID-19 disease. Since patients were referred by clinicians in the hospital, there likely was selection bias that may have been influenced by the clinician or patient/family preference. This selection criteria of the Expanded Access Treatment protocol likely biased our study population towards more severely ill hospitalized COVID-19 patients. In this work, we did not use a comparator but rather focused on making observations of about the antibody profiles only in patients treated in with CP. As discussed in the manuscript, this means our observations may only apply to patients with life threatening COVID-19 inpatients rather than the broader spectrum of COVID-19 illness.
Ethics oversight	The donor plasma procurement protocol and the use of the expanded access protocol was approved by the Albert Einstein College of Medicine Institutional Review Board. Secondary use of de-identified data and biological samples was approved by the MassGeneral Brigham Healthcare Institutional Review Board.

Note that full information on the approval of the study protocol must also be provided in the manuscript.

Flow Cytometry

Plots

Confirm that:

☒ The axis labels state the marker and fluorochrome used (e.g. CD4-FITC).

☒ The axis scales are clearly visible. Include numbers along axes only for bottom left plot of group (a 'group' is an analysis of identical markers).

☒ All plots are contour plots with outliers or pseudocolor plots.

☒ A numerical value for number of cells or percentage (with statistics) is provided.

Methodology

Sample preparation	ADCP:<br>THP-1 cells were grown in culture, incubated with immune complexes containing fluorescent beads conjugated to antigen.<br><br>ADNP:<br>Neutrophils cells were isolated the day of the assay with negative selection (Stemcell) from whole blood, incubated with immune complexes containing fluorescent beads conjugated to antigen, and then stained with an anti-CD66b.<br><br>ADNK:<br>NK cells were isolated the day of the assay with negative selection (RosetteSep - Stem Cell Technologies) from buffy coats, incubated with a staining cocktail containing anti-CD107a BV605 antibody, Golgi stop, and Brefeldin A before being added to ELISA plates with immune complexes already immobilized. Cells were then fixed, stained with anti-CD3 and axnti-CD56 antibodies, permeabilized and intracellularly stained with anti-MIP-1ß and anti-IFNγ antibodies.
Instrument	Intellicyt iQue Screener Plus
Software	iQue Forecyt Standard Edition 8.1 was used to collect and process the data.
Cell population abundance	ADCP:<br>THP-1 cells were grown in culture and confirmed to be uncontaminated.<br><br>ADNP:<br>Neutrophils purity was assessed by CD66b+ cells in the granulocyte gate. The fraction of CD66+ granulocytes was >95% for all donors.<br><br>ADNK:<br>NK purity was assessed by CD56b+and CD3- cells in the granulocyte gate. The fraction of CD56b+and CD3- granulocytes was >90% for all donors.
Gating strategy	ADCP:<br>All events were gated for granulocytes using FSC-H and SSH-H and then single cells using SSC-A and SSC-H. Phagocytic cells were identified in the BL4 channel.<br><br>ADNP:<br>All events were gated for granulocytes using FSC-H and SSH-H and then single cells using SSC-A and SSC-H. Neutrophils were selected as CD66b+ single cells. Phagocytic cells were identified in the BL4 channel.<br><br>ADNK:<br>All events were gated for granulocytes using FSC-H and SSH-H and then single cells using SSC-A and SSC-H. NK were selected as CD56b+ CD3- single cells. Phagocytic cells were identified in the BL4 channel. MIP-1ß + NK cells were identified in the VL1 channel. IFNγ + NK cells were identified in the BL2 channel. CD107a + NK cells were identified in the VL4 channel.

☒ Tick this box to confirm that a figure exemplifying the gating strategy is provided in the Supplementary Information.
