# Supplementary material for: Functional convalescent plasma antibodies and pre-infusion titers shape the early severe COVID-19 immune response
Source: Nat Commun. 2021 Nov 25;12:6853. doi: 10.1038/s41467-021-27201-y (PMC8617042; doi:10.1038/s41467-021-27201-y)
Supplement: Supplementary file 4 — Description of Additional Supplementary Files [file 41467_2021_27201_MOESM4_ESM.pdf]

Description of Additional Supplementary Files

Supplementary Data 1

This file contains all of the processed antigen-specific antibody data described in the main text and Supplementary materials.
